# Highly efficient photonic radar by incorporating MDM-WDM and machine learning classifiers under adverse weather conditions

**DOI:** 10.1371/journal.pone.0300653

**Published:** 2024-04-01

**Authors:** Sushank Chaudhary, Abhishek Sharma, Kuldeep Singh, Sunita Khichar, Jyoteesh Malhotra

**Affiliations:** 1 School of Computer, Guangdong University of Petrochemical Technology, Maoming, China; 2 Department of Electronics Technology, Guru Nanak Dev University, Amritsar, India; 3 Department of Electrical Engineering, Chulalongkorn University, Bangkok, Thailand; 4 Department of Electronics and Communication Engineering, National Institute of Technology, New Delhi, India; Government College University Lahore, PAKISTAN

## Abstract

Photonic radar, a cornerstone in the innovative applications of microwave photonics, emerges as a pivotal technology for future Intelligent Transportation Systems (ITS). Offering enhanced accuracy and reliability, it stands at the forefront of target detection and recognition across varying weather conditions. Recent advancements have concentrated on augmenting radar performance through high-speed, wide-band signal processing–a direct benefit of modern photonics’ attributes such as EMI immunity, minimal transmission loss, and wide bandwidth. Our work introduces a cutting-edge photonic radar system that employs Frequency Modulated Continuous Wave (FMCW) signals, synergized with Mode Division and Wavelength Division Multiplexing (MDM-WDM). This fusion not only enhances target detection and recognition capabilities across diverse weather scenarios, including various intensities of fog and solar scintillations, but also demonstrates substantial resilience against solar noise. Furthermore, we have integrated machine learning techniques, including Decision Tree, Extremely Randomized Trees (ERT), and Random Forest classifiers, to substantially enhance target recognition accuracy. The results are telling: an accuracy of 91.51%, high sensitivity (91.47%), specificity (97.17%), and an F1 Score of 91.46%. These metrics underscore the efficacy of our approach in refining ITS radar systems, illustrating how advancements in microwave photonics can revolutionize traditional methodologies and systems.

## 1. Introduction

Innovative applications of microwave photonics are revolutionizing the transportation sector, particularly in the realm of autonomous vehicle (AV) technology. By enhancing the precision and range of sensing capabilities, microwave photonics plays a crucial role in the advancement of self-driving cars. AV’s, also known as self-driving cars, are capable of sensing their environment and navigating without human input. Autonomous operation of Intelligent Transportation Systems (ITS) requires real-time decision-making, which necessitates the training of ITS using a machine learning (ML) approach [1]. Collecting and processing data from various sensors, then training the ITS with a supervised learning model based on that processed data, is crucial. Thus, it is important to have sensors that can collect data effectively even under challenging atmospheric conditions and low visibility for AV’s operation to detect, classify, and recognize surrounding targets. Radio Detection and Ranging (RADAR) is considered as the main and standard method for target detection, classification, and recognition in all weather conditions [2]. Conventionally, radars is designed with electronic components and hence results in limited functions with poor resolution, limited bandwidth and low speed. Hence to overcome such disadvantages of conventional RADAR, researchers came with Photonics-based technologies to conventional radar system [3]. These technologies were having discrete features of modern photonics such as wider bandwidth, multifaceted multiplexing, rapid signal processing, electromagnetic interference (EMI) immunity, flat response and minimal transmission loss [4–6]. Based upon measurement method of photonic radar is termed as pulsed radar and continuous wave radar. In pulsed radar system, the detection is carried out using time of flight technique which is very effective method in remote sensing and aerial navigation. However, continuous wave radar is preferred which require low input power and light weight equipment due to use of solid-state devices. Continuous wave radar works with signal modulation in amplitude, phase and frequency domain. In this work, we are primarily concentrating on evaluating the performance characteristics of photonic radar systems that operate using Frequency Modulated Continuous Wave (FMCW) technology. FMCW radar offers several benefits, such as a relatively uncomplicated hardware configuration, lower peak power, and the ability to obtain both range and velocity information of targets at the same time [7, 8]. FMCW based photonic radar operates in two configurations viz direct detection and Coherent detection [9]. In direct detection method, backscattered signal is analysed using square law detection scheme and hence the sensitivity of the system is towards intensity of the received signal only. Coherent detection is sensitive to the phase, polarization, and intensity of the incoming signal, as referenced in [10], operating on a principle of linear detection. Coherent systems are further described as homogeneous and heterogeneous. For extended-distance telemetry, the FMCW photonic radar system utilizes the Doppler effect alongside the time of flight method [11]. Further power consumptions of data collecting sensors should be kept low as limited power is available in AV’s. As FMCW based photonic radar employs solid state components hence minimal power is needed for their operation as well as small size gives more flexibility to automobile designer in attaining Low SWaP-C (Size, Weight, Power and Cost) [12]. In 2020 [13], authors have proposed a microwave photonic radar, which employs a post bandwidth synthesis technique, is capable of offering a bandwidth of 16 GHz and a range resolution of 1 cm, according to the authors. In another work [14], The use of wavelength division multiplexing (WDM) and radio over fiber technique has been suggested by authors to create a photonic radar that can generate and distribute radar signals. This proposed microwave photonic radar can achieve a range resolution of 7.3 cm. In another work [15], the authors have shown the capabilities of a linear frequency-modulated continuous-wave photonic radar, which can track the radar cross-section of multiple moving targets even in adverse weather conditions such as fog, cloud, and rain. Furthermore, in real-world traffic scenarios that involve numerous mobile targets, detecting, associating data, and classifying targets become even more difficult tasks. In 2021 [16], The authors have suggested the use of an X-band photonic radar that utilizes a photonic frequency quadrupling scheme for real-time detection of low radar cross-section targets and high-resolution imaging. They have also employed a balanced photo detector and delay interferometer to enhance the signal-to-noise ratio. The proposed photonic radar has demonstrated the ability to detect targets located 2.7 km away from the radar. Recently, In 2022 [9], a comparison was made by authors between the performance of direct detection FMCW-based photonic radar and coherent detection FMCW-based photonic radar. The results indicated that coherent detection-based photonic radar outperformed direct detection FMCW-based photonic radar, but it is more complex and costly. Another study [17] proposed microwave photonic radar that utilizes sparse stepped frequency (SSF) chirp signals to achieve ultra-high resolution. This photonic radar was able to distinguish between two simulated point targets placed 8.3 mm apart. In this proposed work, photonic radar is developed to detect multiple targets under diverse movement of vehicles. For operational ease, linearly frequency modulated continuous wave (L-FMCW) based photonic radar in coherent detection scheme is employed here. LFM chirp is used to modulate the input signal and de-chirping is intelligible mixing of received echo signal with the local oscillator (LO) [10, 18]. The multiple targets detection is accomplished by employing mode division multiplexing (MDM) along with wavelength division multiplexing. MDM improves the security and capacity of the optical system [19]. Four targets are considered in this work and their detections under sever atmospheric conditions is verified. Lastly the data collected using proposed system is further trained using supervised learning model of machine learning. Employing machine learning classifiers like Decision Tree (DT), Extremely Randomized Trees (ERT) and Random Forest (RF), we enhanced the target recognition accuracy. The significant contribution of presented work is as follows:

1. **Robust Performance Evaluation Under Varied Fog Conditions:** Demonstrated the photonic radar system’s efficacy in diverse fog scenarios, including heavy, medium, and light fog, highlighting its adaptability and reliability in different meteorological environments.
2. **Solar Noise Resilience Analysis:** Conducted a thorough assessment of the system’s performance under solar noise influences, particularly strong and weak scintillations, proving its robustness against solar interference, which is critical for real-world ITS applications.
3. **Integration of Advanced Mode division multiplexing-Wavelength division multiplexing (MDM-WDM) and Machine Learning Techniques:** Innovatively combined MDM-WDM with sophisticated machine learning classifiers, significantly enhancing target detection accuracy and recognition in adverse weather and solar conditions.
4. **Benchmarking and Performance Metrics in Adverse Conditions:** Achieved impressive performance metrics such as high accuracy, sensitivity, and specificity, establishing new benchmarks for photonic radar systems in ITS, especially under challenging environmental conditions.

This paper firstly discusses the operation principle of photonic radar which is presented in Section 2. In Section 3 system modelling is presented. Section 4 discusses the findings of proposed model while Section 5 discusses training of the data set attained using proposed model. Section 6 concludes the papers and discusses future directions in this work.

## 2. Operating principal

The efficiency of AV’s is defined by its ability to distinguish between different targets that are closely spaced under adverse atmospheric conditions. This efficiency is termed as range resolution (*L*_*Res*_) and can be expressed arithmetically as in Eq 1 [20]:

$$L_{Res}=\frac{c}{2B}$$ (1)

where *c* is speed of light in free space and *B* is bandwidth of the system. Hence it can be deducted from the above equation that with high bandwidth, resolution will be much narrower and hence more closely spaced targets can be distinguished by the system. The existing LIDAR uses frequency band of 24 GHz which is having very limited bandwidth of 250 MHz also known as Industrial Scientific and medical (ISM) band and thus does not make a suitable candidate for AV applications [21]. Instead of ISM band, short range radar (SRR) band (77 GHz) that offers 4 GHz bandwidth is much suited for AV applications [22]. This higher bandwidth of SSR band offers 20 times improved range resolution and 3 times velocity resolution in comparisons to ISM band. In the proposed photonic radar, Doppler-effect is considered for detection and ranging of targets. Doppler effects have been used in RADARs for target detection. The transmitted radio signal is reflected from the target in terms of echoes and collected by the receiver to extract data such as distance and velocity of the target. The efficacy of the RADAR system is constrained at higher bandwidths in the microwave band due to the diminishing impact of harmonics. Enhancing performance can be achieved by overlaying the microwave signal onto an optical signal. Likewise, RADARs have larger antenna size and high aperture diameters thus offer high beam divergence whereas photonic radar utilizes laser source which is having narrow line-width and low aperture offers low beam divergence and are more suited for AV applications [23].

The frequency of the echo signal received from the target, referred to as the range frequency (*f*_*r*_), is mathematically represented as shown in Eq 2 [24]:

$$f_{r}=\frac{2\times B\times R}{T_{s}\times c}$$ (2)

Where *B* is bandwidth of the system, *R* is range of the target from photonic radar equipped vehicle, *T*_*s*_ is sweep time and *c* is speed of light in vacuum. The system is further tested for adverse atmospheric attenuation and scintillation effects. Lastly the data is utilised for training of autonomous system using supervised learning model.

## 3. System modelling

**Fig 1:**
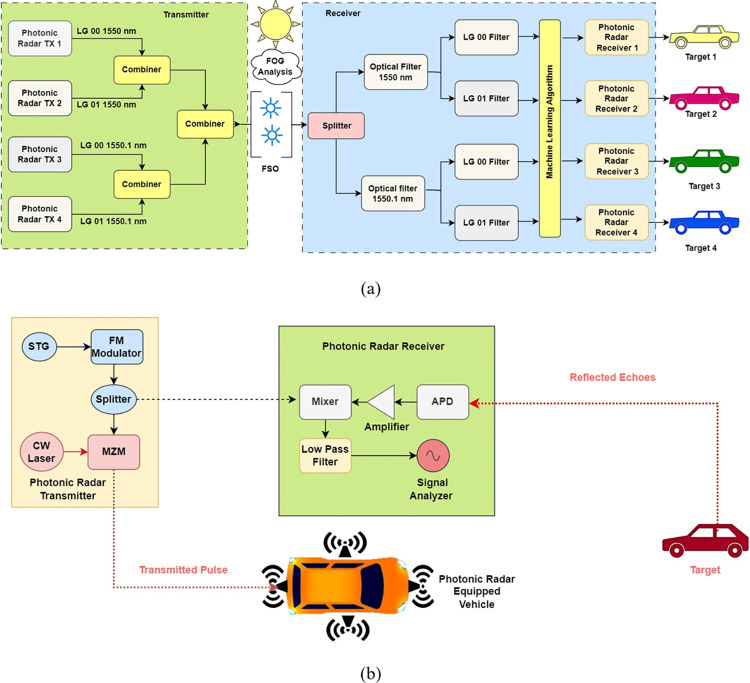
Proposed 4 targets based photonic radar system (a) MDM-WDM Photonic Radar Configuration (b) Photonic Radar Transmitter Configuration.

The modeling of the proposed photonic radar system, which incorporates MDM-WDM, is illustrated in Fig 1 of the manuscript. This system was simulated using OptiSystem^TM^ and MATLAB^TM^ software.

**Fig 2:**
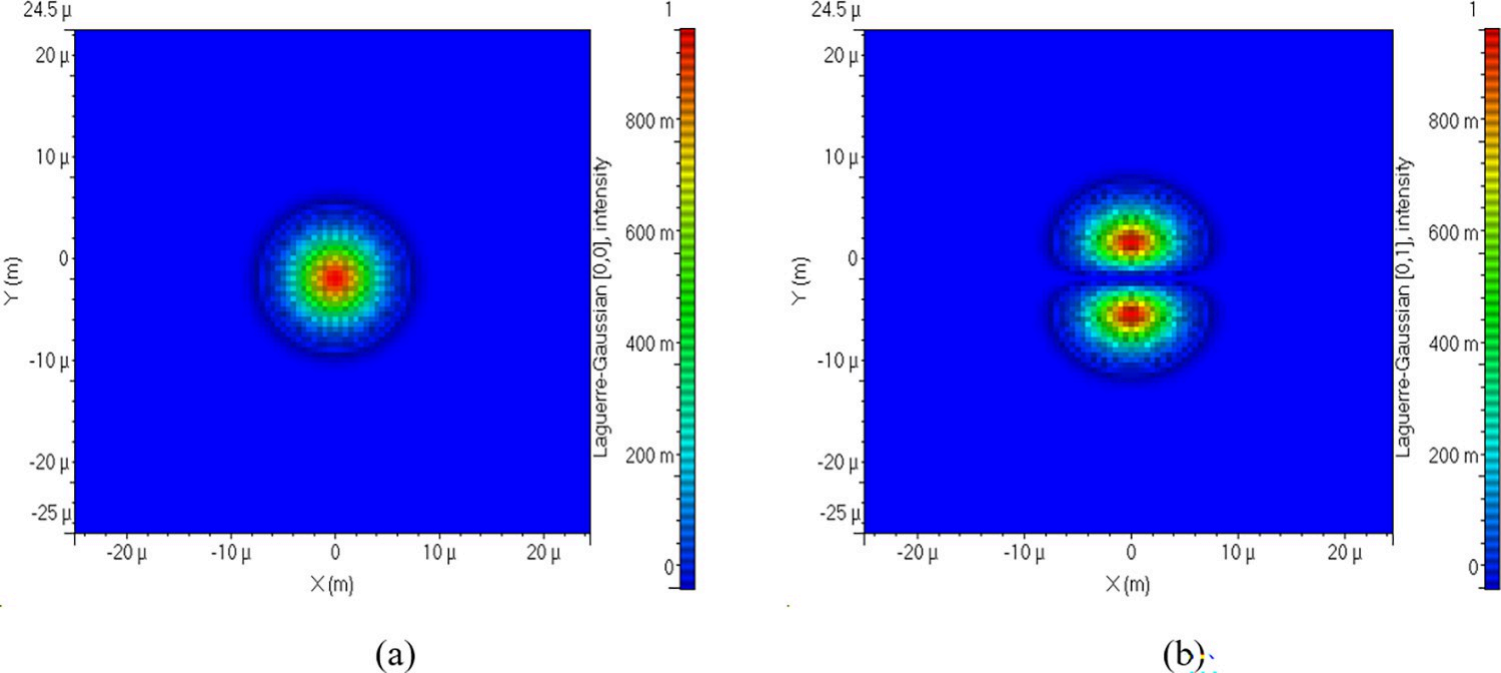
Mode profiles of LG modes (a) LG 00 (b) LG 01.

As shown, four transmitters are transmitting signal over a single channel and two wavelengths that is 1550 nm and 1550.1 nm. Every transmitter contains three sections: Linear Frequency Modulator (LFM), CW source with corresponding mode generator and dual port Mech Zehnder modulator (DMZM) modulator. In LFM section, pseudorandom signal is encoded into triangular waveform. As triangular waveform provides fast sweep rate hence it is chosen over other waveform [25, 26]. This encoded triangular signal is further modulated with microwave frequency in LFM with 77 GHz radio signal having 4 GHz of bandwidth. The radio frequency-modulated LFM signal is divided into two parts; one part is directed to the modulator, while the other serves as a reference signal for the receiver. A continuous wave laser source is used as carrier signal with low input power of -10 dBm. The optical carrier is the fed into transverse mode generator where mode profiles are attached to the optical signal. In this work, we have engaged Laguerre-Gaussian (LG) mode profile and is expressed mathematically as in Eq 3 [27]:

$$\psi_{m,n}(r,\varphi )={\left(\frac{2r^{2}}{w_{o}^{2}}\right)}^{\left|\frac{n}{2}\right|}L_{m}^{n}(\frac{2r^{2}}{w_{o}^{2}})\exp(\frac{r^{2}}{w_{o}^{2}})\exp(j\frac{\pi r^{2}}{\lambda R_{o}^{2}})\{\begin{array}{l} Sin(|n|\varphi ),n\geq 0\\ Cos(|n|\varphi ),n\geq 0 \end{array}\}$$ (3)

where, *m* and *n* represent azimuthal and radial indexes. *L*_*n*,*m*_ represents Laguerre Polynomial, *r* is radius and *w*_*o*_ is spot size. Two modes has been used here that is LG 00 and LG 01 such that channel 1 uses LG 00 and Channel 2 uses LG 01 on same wavelength of 1550 nm. Likewise channel 3 uses LG 00 and channel 4 uses LG 01 on same wavelength of 1550.1 nm. Mode profiles of LG 00 and LG 01 is shown here in Fig 2.

The optical signal, created in a particular mode, undergoes modulation with the LFM signal using a DMZM. The output signal from the DMZM is mathematically expressed as shown in Eq 4 [24]:

$$E(t)=\frac{E_{in}(t)}{10^{IL/20}}\times (\begin{array}{l} (\gamma \times e^{\left(j\pi v_{2}(t\right)/V_{\pi RF}+j\pi v_{bias2}/V_{\pi DC}})+\\ \left((1-\gamma )\times e^{\left(j\pi v_{1}(t\right)/V_{\pi RF}+j\pi v_{bias1}/V_{\pi DC}}\right) \end{array})$$ (4)

where, *E*_*in*_ *(t)* is intensity of optical signal, *IL* represents insertion loss, *V*_*πDC*_ & *V*_*πRF*_ signify switching bias voltage and modulation voltage respectively. Signal from transmitter 1 and 2 is combined with signal of transmitter 3 and 4 using wavelength division multiplexer. This multiplexed signal is further amplified using an optical amplifier. Modeling of the channel, taking into account severe atmospheric conditions, is conducted through a Gamma-Gamma fading channel, and this is depicted mathematically in Eq 5 [28]:

$$P_{Received}=P_{Transmitted}\times \frac{{d_{R}}^{2}}{{\left(d_{T}+\theta R\right)}^{2}}10^{-\alpha \frac{R}{10}}$$ (5)

where, transmitter and receiver aperture diameters are given by *d*_*T*_ & *d*_*R*_ are respectively, beam divergence is given by *θ*, effective distance between transmitter and receiver is given by *R* and atmospheric turbulences are represented by *α*.

For acute atmospheric conditions we have considered fog conditions as visibility reduces drastically with increasing fog thereby affecting the efficiency of AV. Different models has been utilized for calculating attenuation due to rain and fog. Rain attenuation is calculated using Kim model and is expressed in Eq 6 [29]:

$$A_{rain}=k\times {r_{o}}^{\alpha }$$ (6)

where extent of rain in mm/hr is given by *r*_*o*_ while *k* and α are variables calculated by Marshall Palmer distribution [30]. Likewise attenuation due to fog is calculated using Mie scattering model and is mathematically expressed as in Eq 7 [31]:

$$(\lambda )=\frac{3.91}{V}{\left(\frac{\lambda }{550}\right)}^{-\rho }$$ (7)

where scattering coefficient is given by ρ, operational wavelength of system is given by λ and visibility in kilometres is given by *V*. In this work, we have adopted International visibility code [32] as 0.1 dB/km for clear weather and 75 dB/km for heavy fog conditions. The received signal in form of echoes is detected using PIN diode. The power received by PIN diode is given as in Eq 8 [33]:

$$P_{r}=\left\{ \begin{array}{l} P_{t}\frac{\rho_{t}D^{2}\tau_{opt}\tau_{atm}^{2}}{{4R}^{2}}\;for\;extended\;target\\ P_{t}\frac{\rho_{t}{A_{t}D}^{2}\tau_{opt}\tau_{atm}^{2}}{{4R}^{2}A_{ill}}\;for\;any\;target \end{array}\right.$$ (8)

where, *ρ*_*t*_ is target reflectivity, *τ*_*opt*_ and *τ*_*atm*_ the optical and atmospheric transmission loss respectively. *R* is range of target from system, receiver aperture diameter is given by D, while *A*_*t* is_ target area and *A*_*ill*_ is illuminated target area. The photo detector output is mixed with local oscillator signal and the signal is further passed through a low pass filter to obtain beat signal. The beat signal is expressed mathematically as in Eq 9 [9]:

$$S_{b}(t)=R\times A_{lo}\times \sqrt{P_{lo}\times P_{r}}\;\cos\;[2\pi f_{start}\tau +\frac{\pi \beta }{T_{m}}{\left(\tau \right)}^{2}+2\pi f_{r}(t)]\;\sin{[\omega }_{d}(t)+(\theta_{o(t)}-\theta_{lo(t)}$$ (9)

where, amplitude of FM modulator is given by *A*_*lo*_ and input power of low pass filter is given by *P*_*lo*_, ℜ is the responsivity of photo detector, *β* is the modulation index, range frequency is given by *f*_*r*_ and initial frequency is given by *f*_*start*_.

## 4. Results and discussion

**Fig 3:**
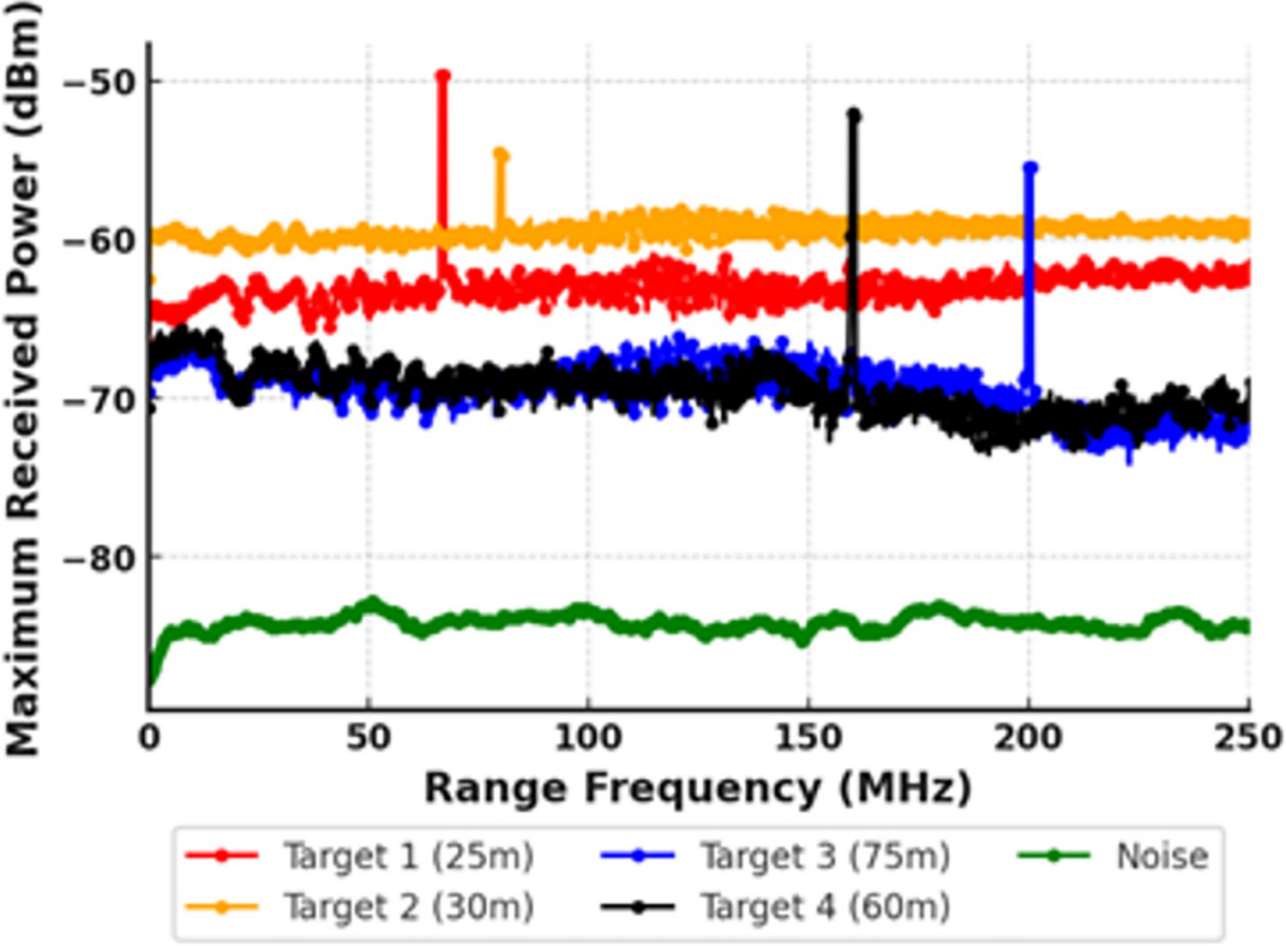
Maximum received power for multi-target detection at varying distances in clear atmospheric condition.

The section discusses simulation results for a proposed MDM-WDM based photonic radar system. Firstly, the system was tested under clear atmospheric conditions, where attenuation was considered to be 0.1 dB/km. The detection of multiple targets under these conditions is depicted in Fig 3.

In the depicted scenario, it is posited that four separate targets are positioned at varying lengths from the photonic radar system: the first target at 25 meters, the second at 30 meters, the third at 75 meters, and the fourth at 60 meters. It is observed that the range frequency for target 1 is at 66.66 MHz, for target 2 it is at 80 MHz, for target 3 it is at 200 MHz, and for target 4 it is at 160 MHz. The range frequencies obtained from the simulation results depicted in Fig 3 match with the theoretically calculated values of range frequency using Eq 2, indicating error-free detection of multiple targets. The proposed photonic radar system is further tested under adverse atmospheric conditions.

**Fig 4:**
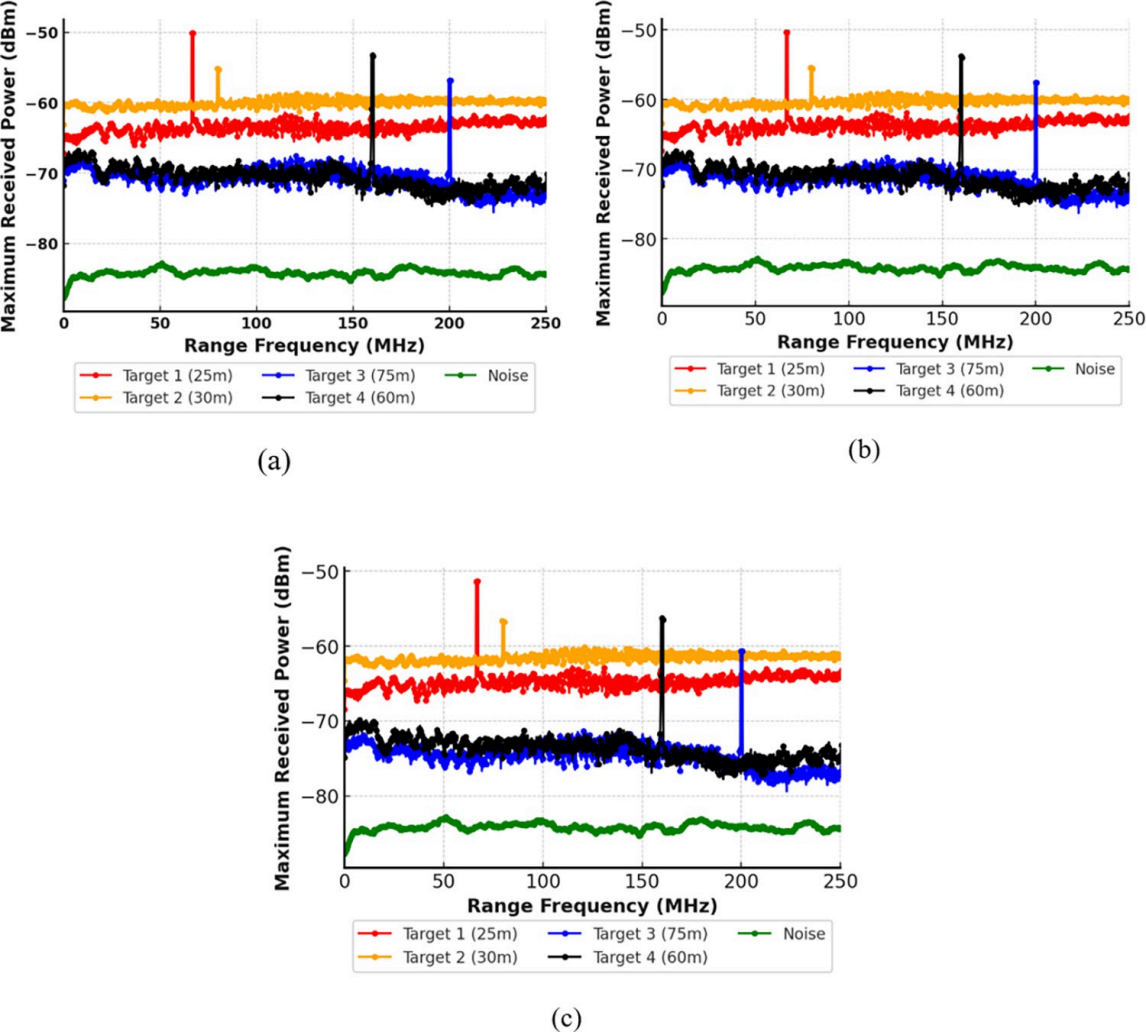
Multiple Target detection under heavy attenuation of (a) Low Fog at 12.5 dB/km (b) Medium Fog at 25 dB/km and (c) Heavy Fog at 75 dB/km.

Firstly, the system is tested for varying fog conditions. According to the international visibility code, three conditions have been considered, namely low fog with an attenuation of 12.5 dB/km, medium fog with an attenuation of 25 dB/km, and heavy fog with an attenuation of 70 dB/km. Fig 4(A) shows the impact of low fog conditions when attenuation is kept at 12.5 dB/km, as per the international visibility code. It can be observed that there is a loss of power when comparing the maximum received power with clear weather conditions. However, successful detection of all four targets has been reported. Similarly, in Fig 4(B), with medium fog conditions, although further power loss is observed, all the targets have been successfully detected. Finally, with heavy attenuation, it was observed that all targets are detected with less power as compared to light and medium fog conditions.

**Fig 5:**
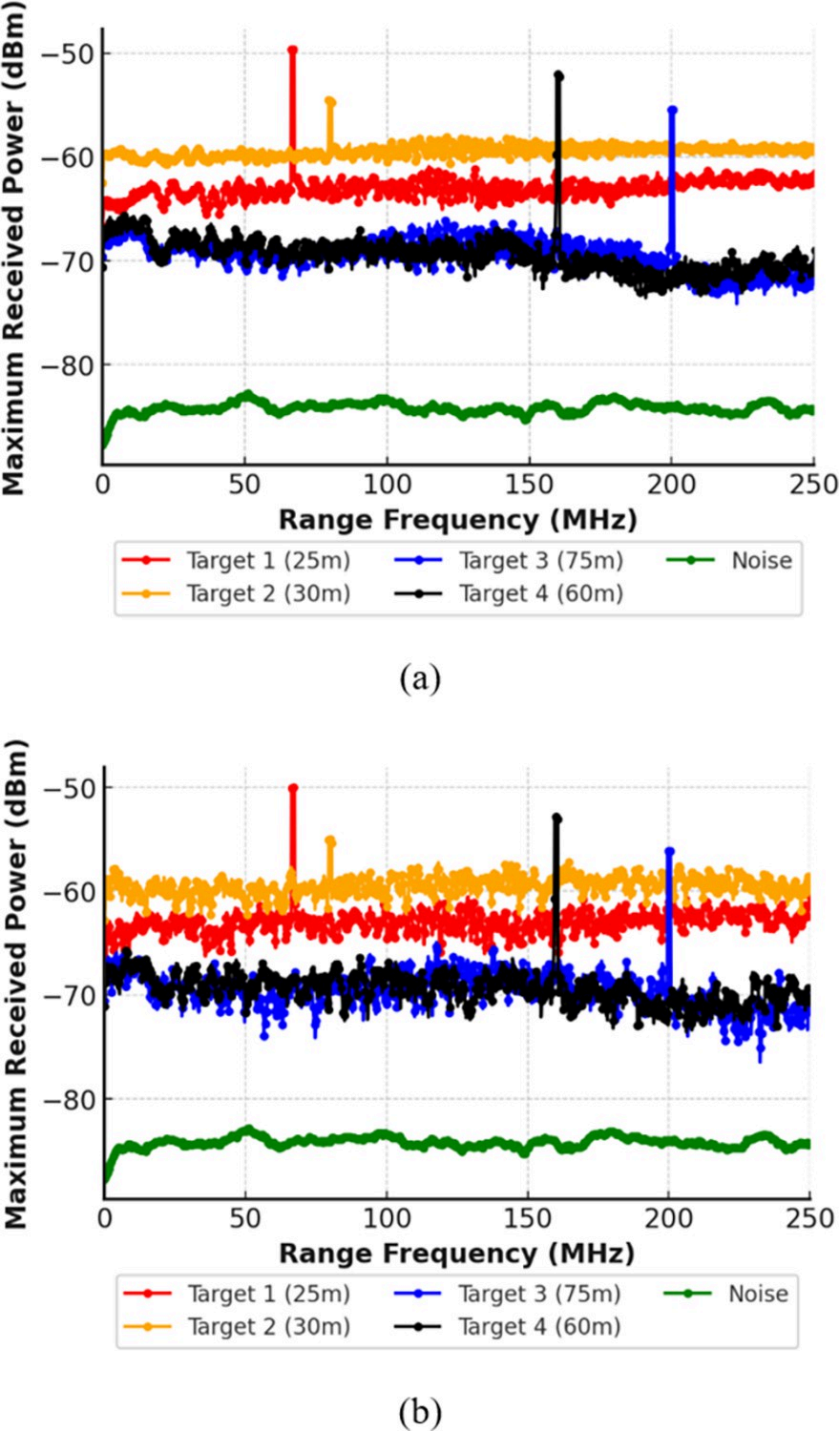
Multiple Target detection under (a) weak scintillations and (b) strong scintillations.

Hence, the results indicate that the suggested model achieves accurate detection in all low, medium and heavy fog conditions. Lastly, the system is tested for the impact of scintillation effects. Two different scenarios are considered, with strong scintillation of 10^−6^ and weak scintillation of 10^−22^. Fig 5 depicts the impact of scintillation on the proposed system. As shown in Fig 5, the scintillation has a minimal impact on the signal intensity, and the results obtained in weak as well as strong scintillation are comparable.

Table 1 shows the measured power levels of all detected targets for different atmospheric conditions. It shows that all the targets have been successfully detected in all atmospheric conditions.

**Table 1:** Measured power values for all targets under different atmospheric conditions.

Atmospheric Conditions	Target	Detected Frequency (MHz)	Power (dBm)
Clear Weather	1	66.66	-49.60
	2	80	-54.47
	3	200	-55.42
	4	160	-52.06
Low Fog	1	66.66	-50.08
	2	80	-55.04
	3	200	-56.84
	4	160	-53.20
Medium Fog	1	66.66	-50.33
	2	80	-55.34
	3	200	-57.59
	4	160	-53.80
Heavy Fog	1	66.66	-51.35
	2	80	-56.57
	3	200	-60.67
	4	160	-56.26
Weak Scintillations	1	66.66	-49.60
	2	80	-54.47
	3	200	-55.42
	4	160	-52.06
Strong Scintillations	1	66.66	-49.98
	2	80	-54.85
	3	200	-56.14
	4	160	-52.85

## 5. Training with ML

In this section, we investigate the efficacy of various machine learning classifiers for enhancing the target detection capability of our photonic radar system. We compared the performance of six classifiers: DT, ERT, RF, Histogram-Based Gradient Boosting (HGB), Extreme Gradient Boosting (XGB), and Adaptive Boosting (AB). DT is known for its simplicity and interpretability, often used in various applications due to its straightforward decision-making process. ERT is a type of ensemble learning technique that combines multiple decision trees to improve prediction accuracy and control over-fitting. RF is another ensemble technique that uses a collection of decision tree classifiers to improve predictive performance and robustness. HBG Boosting is a fast, scalable machine learning algorithm that builds an ensemble of decision trees in a gradient boosting framework. XGB is an efficient and scalable implementation of gradient boosting that is popular for structured or tabular data while AB is a technique that combines multiple weak classifiers to form a strong classifier, adapting by focusing more on instances that previous classifiers misclassified.

Our comparative analysis focused on key performance metrics: accuracy, sensitivity, specificity, F1 Score, and false discovery rate (FDR). A dataset was created for training and testing the model in various conditions, including clear weather, strong and weak attenuation effects, and low, medium, and heavy fog. The dataset includes four target vehicles located at different positions on the road, as shown in Table 2.

**Table 2:** Data summary.

Weather Conditions	Target Vehicles Category
Clear Weather Attenuation: Strong Weak Heavy Fog Medium Fog Low Fog	Target1 Target2 Target3 Target4

Table 2 shows that in 6 different weather conditions, 4 different vehicles have been attempted to be identified simultaneously. Therefore, a total of 24 samples have been generated for training the model, which has 4096 elements of different frequency levels as input. The input elements and output have been represented in numerical form. Similarly, the output vector has been created with 4 class categories of targets, representing Target 1 to Target 4.

**Fig 6:**
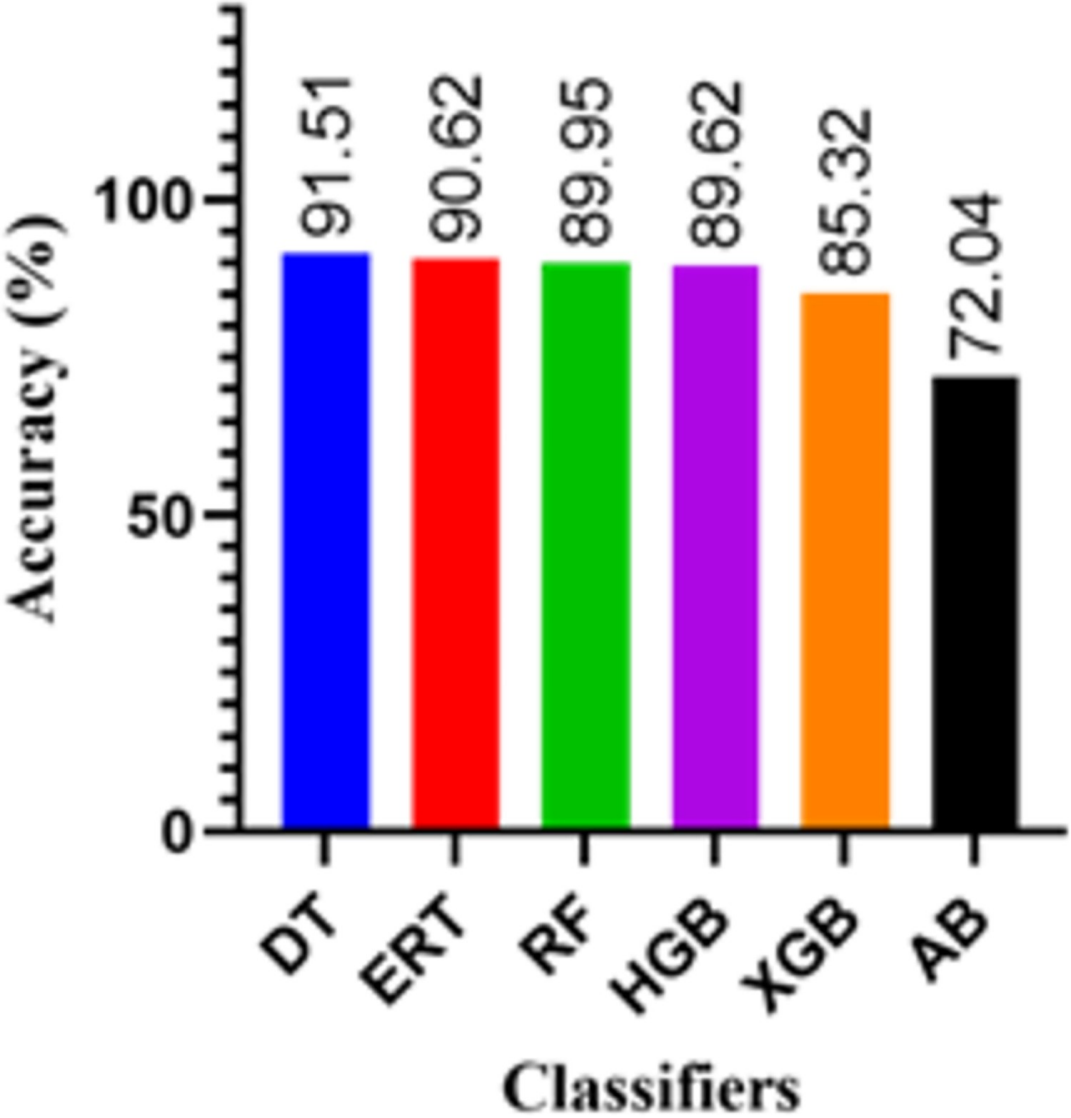
Performance comparison of given ML classifiers in terms of accuracy for target classification.

**Fig 7:**
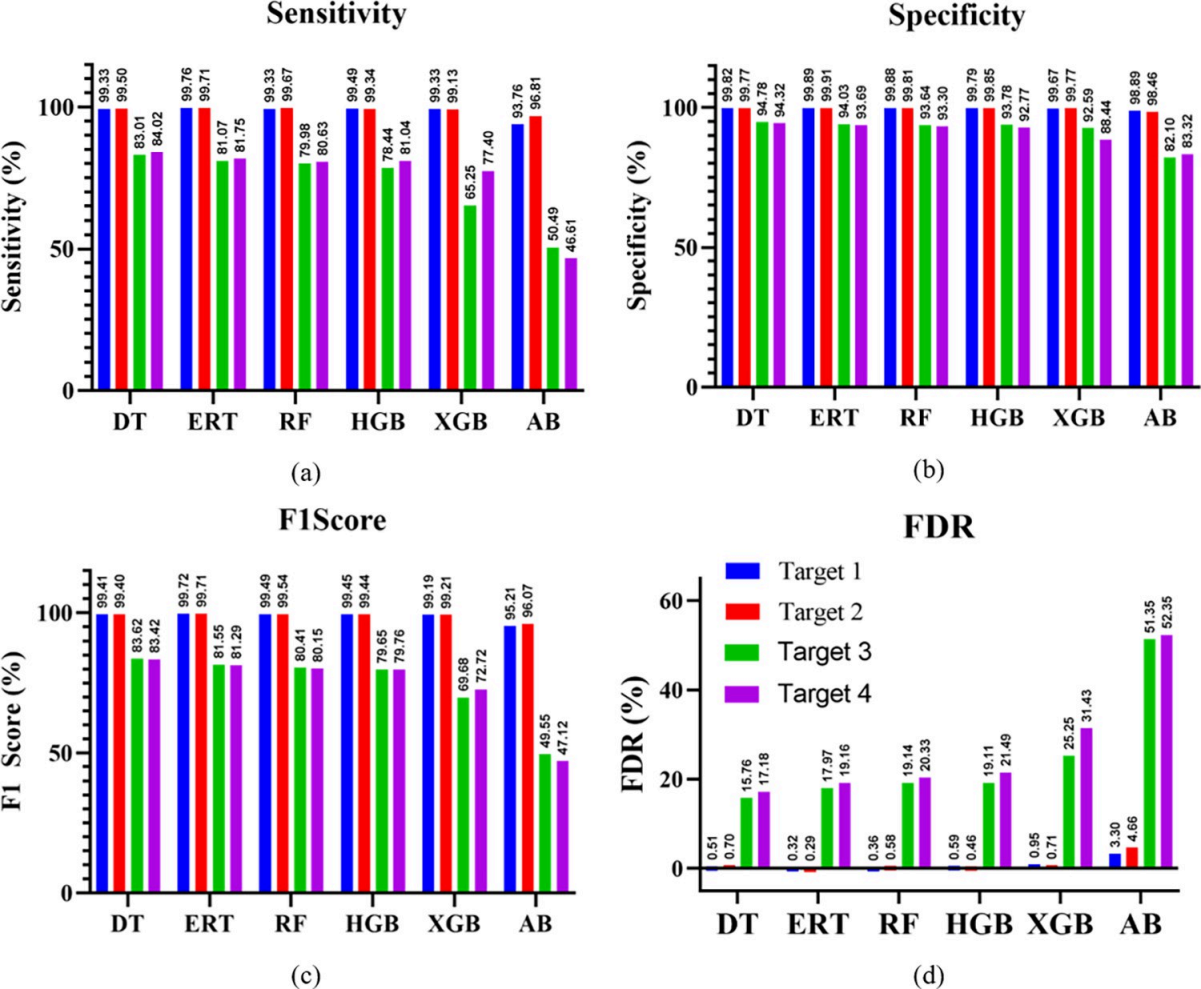
Performance Comparison of given ML classifiers in terms of (a) Sensitivity (b) Specificity (c) F1 Score (d) FDR for different targets.

Figs 6 and 7 depict the performance of proposed system by comparing key performance metrics using different classifiers based upon inputs of Table 2.

Fig 6 shows the overall accuracy of each classifier. Among the classifiers, the Decision Tree stands out with the highest accuracy of 91.51%, indicating its robustness in correctly identifying targets under various conditions. This high accuracy demonstrates the effectiveness of the DT classifier for the photonic radar system in terms of overall target classification accuracy.

Fig 7 provides a detailed analysis of the performance of the given machine learning classifiers in terms of sensitivity, specificity, F1 score, and FDR values for different targets. In Fig 7(A), the DT and ERT classifiers demonstrate high sensitivity, with the Decision Tree achieving a sensitivity of 91.47%. This suggests their effectiveness in correctly identifying true targets, which is vital for avoiding missed detections in radar applications. Fig 7(B) assesses the classifiers’ ability to correctly identify non-targets (true negatives) in terms of specificity. The DT shows high specificity of 97.17%, indicating a lower false alarm rate, crucial for radar systems to avoid unnecessary responses to non-target objects. Similarly, Fig 7(C) highlights the F1 Score, with the Decision Tree achieving a high score of 91.46%, indicating a balanced and effective performance in both precision and sensitivity aspects. Moreover, Fig 7(D) presents the FDR, with the Decision Tree maintaining a relatively low FDR of 8.54%, indicating a higher reliability of the detections made by the classifier. The results of the proposed method, as demonstrated by the DT classifier, are compared with other methods to highlight its superior performance across multiple parameters. This comparison underscores the potential of the Decision Tree classifier as a reliable tool for our photonic radar system, particularly beneficial for precise target detection and classification. The incorporation of the DT classifier into our photonic radar system significantly enhances its target detection proficiency, aligning with our goal of developing a highly accurate and efficient system. Future work will explore the integration of these classifiers in real-world scenarios, further validating their effectiveness in operational environments.

## 6. Conclusion

In this study, we have showcased the innovative applications of microwave photonics through the development of a MDM-WDM based photonic radar system, specifically tailored for advancements in intelligent transportation. Our approach involved rigorous numerical simulations for detecting four distinct targets under various atmospheric conditions, both clear and adverse. The results demonstrated successful target detection, effectively aligning with the theoretical range-frequency predictions and highlighting the strengths of microwave photonics in practical applications. Significantly, the integration of machine learning, particularly using classifiers like DT, ERT, and RF, has markedly enhanced target classification. Achieving an impressive accuracy of 91.51% with high sensitivity and specificity, this integration exemplifies the synergy between advanced photonics and artificial intelligence. This marks a substantial improvement over the previously reported SVM-based model accuracy. As we look to the future, our focus will include expanding the dataset for further training enhancement and implementing real-time test beds. These efforts aim to validate and refine our model in operational scenarios, further demonstrating the transformative impact of microwave photonics in intelligent transportation systems.

## Supporting information

S1 File (ZIP)
